# ESBL-Producing *Escherichia coli* from Cows Suffering Mastitis in China Contain Clinical Class 1 Integrons with CTX-M Linked to IS*CR1*

**DOI:** 10.3389/fmicb.2016.01931

**Published:** 2016-11-30

**Authors:** Tariq Ali, Sadeeq ur Rahman, Limei Zhang, Muhammad Shahid, Shiyao Zhang, Gang Liu, Jian Gao, Bo Han

**Affiliations:** ^1^Department of Clinical Veterinary Medicine, College of Veterinary Medicine, China Agricultural UniversityBeijing, China; ^2^College of Veterinary Sciences and Animal Husbandry, Abdul Wali Khan University, Garden CampusMardan, Pakistan

**Keywords:** *E. coli*, ESBLs, CTX-M-15, integrons, gene cassettes, bovine mastitis

## Abstract

The prevalence of pathogenic multi-drug resistant (MDR) extended-spectrum β-lactamase (ESBL)-producing *Escherichia coli* is rapidly increasing, becoming a global concern. In a veterinary context, ESBL-producing *E. coli* are mostly reported in poultry and pigs. Here, we report on the prevalence and characterize ESBL-producing *E. coli* isolated from diverse dairy farms in China. Overall, 36 (23.53%) out of 153 *E. coli* isolates from mastitic milk samples (*n* = 1252) were confirmed as ESBL-producers by double-disc synergy testing and PCR. Nucleotide analysis of PCR amplicons revealed that *bla*_CTX-*M*_ was the predominant ESBL gene detected in 28 (77.78%) isolates, with *bla*_CTX-*M*-15_ being the major (78.57%) allele encoding for ESBLs. Also, 20 (55.56%) and 6 (16.67%) of the ESBL isolates were carrying *bla*_TEM_ and *bla*_SHV_ genes, respectively, in singlet or in combination. The majority of these isolates belonged to phylo-group A (69.44%) and D (16.67%). Strikingly, all these isolates were found to be MDR showing high resistance to cephalosporins including the fourth generation cefepime and common non β-lactams. Additionally, class 1 integrons (*intI1*) were found in 30 (83.33%) isolates. Analysis of the class 1 integrons variable regions indicated that they were carrying up to five different gene cassettes conferring resistance to various drugs with a predominant combination of *dfrA17-aadA5* genes in tandem, conferring resistance to aminoglycosides and trimethoprim. However, no ESBL encoding genes were found in the cassettes. Interestingly, 22 (66.11%) of the ESBL isolates were also carrying insertion sequence common region 1 (IS*CR1*) which was found to be associated with most of the CTX-M genes. Altogether, the current study reports on the high prevalence of ESBL-positive *E. coli*, particularly CTX-M-15, carrying clinical class 1 integrons and IS*CR1* elements are likely indicative of their rapid and wider dissemination, posing threats to veterinary and public health. To the best of our knowledge, this is the first comprehensive study to report on the alarming high occurrence of ESBL-producing *E. coli* from mastitic cows in China.

## Introduction

Bovine mastitis, inflammation of the mammary gland, is the most prevalent and economically important disease of dairy animals (Halasa et al., 2007). Mastitis can be caused by a variety of bacterial pathogens, but *Escherichia coli* is one of the leading causes (Dahmen et al., 2013). Antimicrobial agents are used for therapeutic as well as preventive measures against bacterial infections including bovine mastitis in farm animals. Beta-lactams, such as ampicillin and amoxicillin, remain the first-line treatment in veterinary medicine but an increase in drug-resistance to these antibiotics has been observed. Therefore, extended-spectrum cephalosporins (ESC) such as ceftiofur have been approved in China for the treatment of animal diseases (MAO, 2010). Unfortunately, several recent studies have reported the increasing occurrence of highly resistant extended-spectrum β-lactamase (ESBL)-producing *Enterobacteriaceae*, mainly *E. coli*, isolated from food-producing animals from various countries including China (Rao et al., 2014; Xu et al., 2015; Seni et al., 2016).

Bacterial resistance to β-lactams, popular antibiotics due to their proven safety and efficiency, is increasing at an alarming rate. This resistance is mainly achieved through β-lactamases that can hydrolyse most β-lactam antibiotics including the third and fourth generation ESCs and monobactams (Bush and Jacoby, 2010). ESBLs are predominantly produced in gram negative bacteria, particularly in *E. coli*, and are considered a key mechanism conferring resistance to cephalosporins (Perez et al., 2007). Multi-drug resistance (MDR) has been commonly observed in most ESBL-producers and more alarmingly, co-resistance to other commonly used antibiotics like aminoglycosides, fluoroquinolones, tetracycline has been often reported (Chen et al., 2010; Timofte et al., 2014; Xu et al., 2015). This renders these organisms resistant to a wide range of antibiotics with limited therapeutic options. ESBL encoding genes have been categorized into three main types: *bla*_CTX-*M*_, *bla*_SHV_, and *bla*_TEM_. The *bla*_CTX-*M*_ has been further categorized into five sub-groups (*bla*_CTX-*M*-1_, *bla*_CTX-*M*-2_, *bla*_CTX-*M*-8_, *bla*_CTX-*M*-9_, *bla*_CTX-*M*-25_) and more than 150 variants have been documented (http://www.lahey.org/studies). In the past few years, CTX-M, especially CTX-M-15, has emerged as the most dominant type of ESBLs globally (D'Andrea et al., 2013). Recently, CTX-M-15 producing *E. coli* have been frequently documented from various sources including humans and food producing animals (Timofte et al., 2014; Liu et al., 2015; Xu et al., 2015), showing the broad spectrum of reservoirs carrying and spreading these genes. Food-animals are well established reservoirs of ESBL-producing *E. coli*, which can be transmitted from animals to humans by various direct and indirect means (Dahmen et al., 2013; Geser et al., 2015). This is also verified by Madec et al. (2012), they reported that the plasmids carrying CTX-M-15 genes in *E. coli* isolated from cattle were highly similar to those found in ESBL-producing *E. coli* isolates from human beings.

Integrons are genetic elements that play a vital role in the development and dissemination of MDR in clinical isolates due to their ability to capture, integrate and express gene cassettes (Vinue et al., 2008; Chen et al., 2010). Three main classes of integrons (1–3), carrying the gene cassettes encoding for antimicrobial resistance genes, are generally found to be associated with antibiotic resistance genes in pathogenic *E. coli*, Class 1 integrons are the most common in clinical *E. coli*, followed by less frequent class 2 integrons (Vinue et al., 2008; Xu et al., 2015). Class 1 integrons contain a 5′ conserved segment (CS) and 3′CS, followed by a variable region that contains one or more gene cassettes. The 5′CS consists of an integrase gene (*intI1*), a recombination-site (*attI1*), and the Pc promoter(s), and the 3′CS includes *qacE*Δ*1* and *sul1* genes which encode for quaternary ammonium compound and sulphonamide resistance (Hall and Stokes, 1993). Moreover, insertion sequences like IS*CR1* (insertion sequence common region 1) as part of the complex class 1 integrons are found to be associated with ESBL and other resistance encoding genes and are probably involved in their mobilization and transposition. IS*CR1* may mobilize the truncated 3′CS and nearby sequences from one integron to the 3′CS of another integron utilizing rolling circle transposition, thus facilitating dissemination of resistance elements (Eckert et al., 2006; Toleman et al., 2006).

Limited studies, particularly from China, have characterized ESBL-producing *E. coli* isolated from diseased food producing animals, mainly from mastitic cows (Lu et al., 2010; Timofte et al., 2014). Thus, we designed the current study to investigate the prevalence of pathogenic ESBL-producing *E. coli* and to characterize the ESBL genes and genetic elements which are likely to be responsible for their mobility and dissemination. To the best of our knowledge, this is the first comprehensive study into the molecular characterization of ESBL genes in *E. coli* isolated from dairy cows in China.

## Materials and methods

### Statement of ethics

The present study was conducted in accordance with the ethical guide lines of China Agricultural University (CAU), Beijing. Proper ethical approval was granted by the departmental committee of College of Veterinary Medicine, CAU. Sampling was carried according to the standard protocols and with prior consent of the dairy herd's authority.

### Sample collection and location

**Figure 1:**
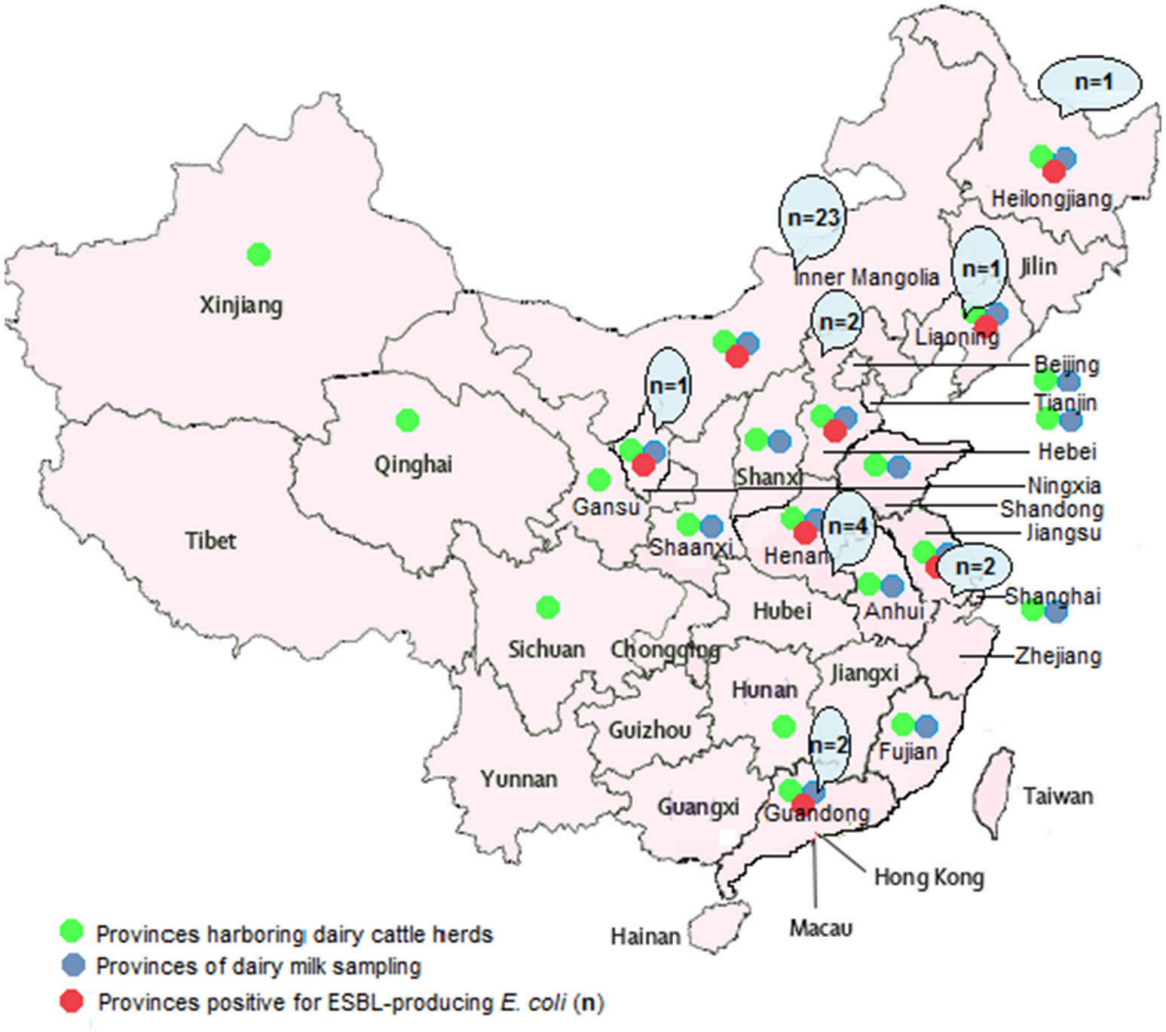
**Map of China (mainland) showing 16 provinces from where samples were collected**.

Milk samples of mastitic cows (*n* = 1252) were collected from 61 large commercial dairy herds (2000–40,000 cows/herd) located in 16 provinces of China during January 2015 to May 2016 (Figure 1 and Table 1). Sampling was carried out when the cows were suffering from mastitis and not according to a fixed schedule. The guidelines of the National Mastitis Council (NMC, 1999) were followed for the collection of milk samples from cows. Samples were taken in 50 mL sterile tubes and transported on ice to the laboratory for further processing.

**Table 1:** **Occurrence of ESBL-producing ***E. coli*** isolated from dairy herds located in 16 provinces of China**.

**Provinces of sampling**	**No. of dairy herds^a^**	**No. of milk samples^b^**	***E. coli* isolates^c^**	**ESBL *E. coli* from each herd^d^**
Anhui	A	63	4	0
Beijing	B/B1/B2	26 (6/9/11)	5 (2/2/1)	0/0/0
Fujian	F	12	0	0
Guangdong	G/G1	98 (23/75)	3 (1/2)	2 (0/2)
Hebei	Hb/ Hb1/ Hb2/ Hb3	220 (11/10/16/16	36 (2/0/0/5	2 (0/0/0/1
	/Hb4/Hb5/Hb6/Hb7 /Hb8/Hb9/Hb10	/6/11/12/18/23 /24/12/38/14/9)	/1/0/0/4/5 /3/3/6/6/1)	/0/0/0/0/1 /0/0/0/0/0)
	/Hb11/Hb12/Hb13			
Heilongjiang	H/H1/H2/H3	73 (10/13/10/40)	7 (4/2/0/1)	1 (1/0/0/0)
Henan	Hn/Hn1/Hn2/Hn3/Hn4	43 (12/6/7/12/6)	5 (2/0/0/2/1)	4 (2/0/0/2/0)
Inner-Mongolia	I/I1/I2/I3/I4/I5	425 (17/14/12/18	45 (1/2/0/1	23 (0/0/0/0
	/I6/I7/I8/I9/I10	/42/37/49/22/24	/6/8/7/5/3	/5/7/6/3/0
	/I11/I12/I13	/61/33/53/22/21)	/6/0/3/2/1)	/2/0/0/0/0)
Jiangsu	J	9	4	2
Liaoning	L	20	4	1
Ningxia	N/N1/N2/N3	97 (15/32/20/30)	19 (2/7/5/5)	1 (0/1/0/0)
Shaanxi	Sx-Bj	13	2	0
Shandong	S/S1/S2/S3	83 (14/14/15/40)	8 (0/1/5/2)	0/0/0/0
Shanxi	Sx-Cz	6	0	0
Shanghai	S/S1/S2/S3	59 (16/16/10/17)	10 (1/0/6/3)	0/0/0/0
Tianjin	T	5	1	0
Total	61	1252	153 (12.22%)	36 (23.53%)

a The letters in the column represent the farm number.

b The numbers in parenthesis indicate no. of milk samples corresponding to the respective farm in second column.

c The numbers in parenthesis show E. coli isolates from the respective farm.

d The numbers in parenthesis indicate ESBL-producing E. coli isolated from the respective farm.

### Isolation and identification of *E. coli*

Milk samples, shortly after arrival, were streaked (10 μL) onto MacConkey Agar (Difco™, Becton Dickinson, Sparks, MD USA) and incubated at 37°C for 18–24 h. Presumptive *E. coli* colonies with the dark pink to red colors, were further confirmed with the API-20E kit (bioMérieux, Marcy I'Etoile, France) as per instruction of the manufacturer. Biochemically confirmed *E. coli* isolates were further verified by PCR as described previously (Tantawiwat et al., 2005). Confirmed *E. coli* isolates were stored in brain heart infusion broth (BHI; Sigma-Aldrich) containing 30% glycerol at −80°C.

### Phenotypic screening of ESBL-Producers

*E. coli* isolates were first screened for the phenotypic identification of ESBLs-producers on MacConkey agar containing cefotaxime (1 mg/L). These presumptive ESBL-producing *E. coli* were further confirmed by double-disc synergy testing in accordance with recommendations of the Clinical and Laboratory Standards Institute (CLSI, 2014), using antimicrobial discs (Becton Dickinson, Sparks, MD USA) of cefotaxime (30 μg), cefotaxime plus clavulanic acid (30/10 μg), ceftazidime (30 μg), and ceftazidime plus clavulanic acid (30/10 μg). The test was recorded positive when the zone of inhibition of cefotaxime plus clavulanic acid or ceftazidime plus clavulanic acid was ≥5 mm larger than their respective single discs (CLSI, 2014).

### Genotypic screening of ESBL-Producing *E. coli* isolates

Bacterial DNA from ESBL-positive *E. coli* was isolated by the TIANamp Bacteria DNA Kit (TIANGEN, Beijing, China) according to the manufacturer's instructions. PCR assays were used for the detection of *bla*_CTX-*M*,_*bla*_SHV_, *bla*_TEM_ genes as described previously (Chen et al., 2010). Details of the primers used in this study are shown in Table 2. All ESBL genes relevant PCR amplicons were purified by the TIANquick Midi Purification Kit (TIANGEN, Beijing, China), bi-directionally sequenced and aligned with sequences available in GenBank (Chen et al., 2010). *Klebsiella pneumoniae* ATCC 700603 (ESBLs-positive strain) and ddH_2_O, instead of template DNA, was used as positive and negative controls, respectively, in all PCR assays.

**Table 2:** **Details of primers used in this study**.

**Primers**	**Sequence (5′ to 3′)**	**Target gene**	**Annealing temperature**	**Amplicons size**	**References**
β**-lactamases**					
CTX-MA CTX-MB	CGC TTT GCG ATG TGC AG ACC GCG ATA TCG TTG GT	*bla*_CTX-*M*_	54°C	550-bp	Villegas et al., 2004
SHV-F SHV-R	GGG TTA TTC TTA TTT GTC GC TTA GCG TTG CCA GTG CTC	*bla*_SHV_	58°C	567-bp	Chang et al., 2001
TEM-F TEM-R	ATA AAA TTC TTG AAG ACG AAA GAC AGT TAC CAA TGC TTA ATC	*bla_TEM_*	56°C	1086-bp	Yao et al., 2007
**INTEGRONS**					
intI1-F	CCT CCC GCA CGA TGA TC	*intI1*	54°C	280-bp	Dillon et al., 2005
intI1-R	TCC ACG CAT CGT CAG GC				
intI2-F	AAA TCT TTA ACC CGC AAA CGC	*intI2*	54°C	439-bp	Dillon et al., 2005
intI2-R	ATG TCT AAC AGT CCA TTT TTA AAT TCT A				
intI3-F	AGT GGG TGG CGA ATG AGT G	*intI3*	54°C	599-bp	Dillon et al., 2005
intI3-R	TGT TCT TGT ATC GGC AGG TG				
intI1-VR-F	TCA TGG CTT GTT ATG ACT GT	*intI1*variable region	56°C	variable	White et al., 2000
ntI1-VR-R	GTA GGG CTT ATT ATG CAC GC				
***E. coli*****-SPECIFIC**					
UAL	TGG TAA TTA CCG ACG AAA ACG GC	*uidA*	62°C	147-bp	Tantawiwat et al., 2005
UAR	ACG CGT GGT TAC AGT CTT GCG				
**PHYLO-GROUPS**					
ChuA-F	GAC GAA CCA ACG GTC AGG AT	*ChuA*	55°C	279-bp	Clermont et al., 2000
ChuA-R	TGC CGC CAG TAC CAA AGA CA				
YjaA-F	TGA AGT GTC AGG AGA CGC TG	*YjaA*	55^o^C	211-bp	Clermont et al., 2000
YjaA-R	ATG GAG AAT GCG TTC CTC AAC				
TspE4C2-F	GAG TAA TGT CGG GGC ATT CA	*TspE4C2*	55°C	152-bp	Clermont et al., 2000
TspE4C2-R	CGC GCC AAC AAA GTA TTA CG				
**IS*****CR1***					
ISCR1-F ISCR1-R	CGC CCA CTC AAA CAA ACG GAG GCT TTG GTG TAA CCG	IS*CR1*	55°C	469-bp	Kiiru et al., 2013

F, forward; R, reverse.

### Phylogenetic grouping

ESBL-positive *E. coli* isolates were placed in one of the four phylogenetic groups: phylo-group A, group B1, group B2 or group D. For this purpose, a triplex PCR assay targeting the *chuA* and *yjaA* genes and *TspE4* was used as described previously by Clermont et al. (2000). The primers sequences and the annealing temperatures are listed in Table 2.

### Antibiotic susceptibility testing

Antibiotic susceptibility of ESBL isolates was carried out on Mueller-Hinton agar (Difco™) against 16 different antibiotics discs (Becton Dickinson, Sparks, MD, USA), using the standard Kirby-Bauer disk diffusion method according to recommendations of the CLSI (2014). The panel of antimicrobial agents consisted of both β-lactam and non-β-lactam antibiotics as listed in Table 3. *E. coli* ATCC 25922 (ESBL-negative strain) and *K. pneumoniae* ATCC 700603 (ESBL-positive strain) were used as quality control strains (CLSI, 2014). The isolates were declared as multi-drug resistant (MDR) when found resistant to three or more categories of antimicrobial drugs.

**Table 3:** **Antibiotic susceptibility profiles of ESBL-producing ***E. coli*** isolates (***n*** = 36) from milk of mastitic cows**.

**Antimicrobial agents**	**Abbreviations**	Conc.^*^ (μg)	**Susceptible (%)**	**Intermediate (%)**	**Resistance (%)**
Ampicillin	AM	10	11.11 (04/36)	02.78 (01/36)	86.11 (31/36)
Amoxicillin/clavulanic acid	AMX/CA	20/10	25.00 (9/36)	11.11 (04/36)	63.89 (23/36)
Cefalexin	CX	30	00.00 (00/36)	00.00 (00/36)	100 (36/36)
Cefaclor	CEC	30	05.56 (02/36)	00.00 (00/36)	94.44 (34/36)
Cefoxatin	FOX	30	83.34 (30/36)	08.33 (03/36)	8.33 (3/36)
Cefotaxime	CTX	30	00.00 (00/36)	00.00 (00/36)	100.0 (36/36)
Ceftazidime	CAZ	30	33.33 (12/36)	00.00 (00/36)	66.67 (24/36)
Cefepime	FEP	30	41.67 (15/36)	11.11 (04/36)	47.22 (17/36)
Aztreonam	AZT	30	13.89 (05/36)	00.00 (00/36)	86.11 (31/36)
Meropenem	MPN	10	100.0 (36/36)	00.00 (00/36)	00.00 (00/36)
Tetracycline	TE	30	16.67 (06/36)	11.11 (04/36)	72.22 (26/36)
Gentamicin	G	10	27.78 (10/36)	11.11 (04/36)	61.11 (22/36)
Ciprofloxacin	CIP	05	55.56 (20/36)	08.33 (03/36)	36.11 (13/36)
Chloramphenicol	C	30	47.22 (17/36)	11.11 (4/36)	41.67 (15/36)
Nalidixic acid	NAL	30	19.44 (07/36)	02.78 (01/36)	77.78 (28/36)
Trimethoprim/sulphamethoxazole	STX	1.25/23.75	25.00 (09/36)	02.78 (01/36)	72.22 (26/36)

* Conc: concentrations.

### Detection of integrons, gene cassettes and IS*CR1*

A PCR assay was used to detect Class 1, 2, and 3 integrons in all ESBL-producing *E. coli* using integron-integrase gene specific primers, *intI1, intI2*, and *intI3*, respectively (Dillon et al., 2005). Subsequently, the *intI1* positive genotypes (*n* = 24) were determined by sequencing amplicons derived from PCR for the class 1 integron variable regions as described previously (White et al., 2000). The IS*CR1* region was PCR amplified from ESBL-producing isolates using specific primers (Table 2). The sequenced amplicons of the IS*CR1* elements were confirmed by BLAST analysis (see below). A combination of primers specific to the IS*CR1* elements (Kiiru et al., 2013) elements and consensus primers of ESBL genes were used to verify their association.

### PCR-RFLP genotyping of Class 1 integron variable region amplicons

A PCR-based restriction fragment length polymorphism (PCR-RFLP) assay was adopted to identify genetic variation in the amplified products using restriction enzyme *HinfI* (Takara, Shiga Japan) as published previously (Gu et al., 2008). PCR-RFLP products with similar band profiles were regarded as the same genotypes carrying identical gene cassette(s).

### Nucleotide sequencing and data analysis

Regardless of the similar PCR-RFLP genotypes, all amplicons of gene cassettes, IS*CR1* elements, and ESBL genes were bi-directionally sequenced using ABI 3730 sequencer (Applied Biosystems, Foster City, CA, USA). PCR amplicons of >1.8 kb were further sequenced using primer walking based on the sequenced amplicons. The obtained sequences were subjected to BLAST homology searches in the INTEGRALL database (http://integrall.bio.ua.pt). Other sequence analyses were compared with BLASTN software (http://www.ncbi.nlm.nih.gov/BLAST/). Clone Manager 7 (Sci-Ed software, Denver, USA) and ClustalW2 (http://www.ebi.ac.uk/Tools/msa/clustalw2/) were also used for detailed analysis such as alignments and open reading frames.

## Results

### Prevalence and characterization of ESBL-Producing *E. coli*

Overall, 153 *E. coli* isolates were recovered from 1252 milk samples of mastitic dairy cows from 16 different provinces of China. Thirty six (23.53%) isolates were detected as ESBL-producing *E. coli* by phenotypic confirmatory tests and this was also verified by ESBL genotype specific PCR assay. The distribution of these isolates among different cattle herds is shown in Table 1. The highest occurrence of ESBL producers was observed in the Inner Mongolia province (23 isolates), followed by the Henan region (four isolates).

**Figure 2:**
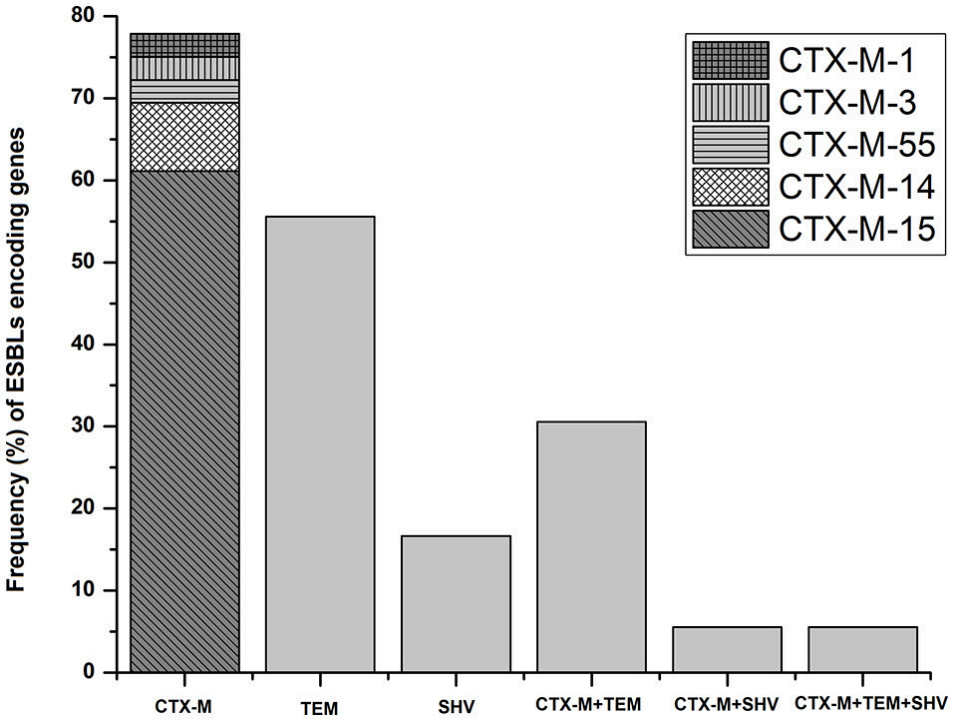
**Distribution of ESBLs encoding genes and CTX-M subtypes among ESBL-producing ***E. coli*** (***n*** = 36) isolated from bovine mastitis**.

Figure 2 shows the frequency (%) of various ESBL encoding genes among 36 ESBL-producing *E. coli* isolated from mastitic milk. Overall, *bla*_CTX-*M*_ was the most prevalent ESBL gene (77.78%; 28/36), while *bla*_TEM_ and *bla*_SHV_ genes were present in 55.56% (20/36) and 16.67% (6/36) of ESBL-positive isolates, respectively. The *bla*_TEM_ and *bla*_SHV_ genes were most frequently observed together with *bla*_CTX-*M*_, rather than alone (Figure 2). Notably, two of the isolates from Inner Mongolia carried three β-lactamase genes (*bla*_CTX-*M*-15_+*bla*_TEM-1_+*bla*_SHV-12_) in combination. Sequence analysis revealed that *bla*_CTX-*M*-15_ was the dominant (78.57%; 22/28) subtype. The other *bla*_*CTX-M*_subtypes were: *bla*_CTX-*M*-14_ (10.71%; 3/28), *bla*_CTX-*M*-1_ (3.57%; 1/28), *bla*_CTX-*M*-3_(3.57%; 1/28), and *bla*_CTX-*M*-55_ (3.57%; 1/28). The phylo-group A was the most prevalent (69.44%; 25/36) among 36-ESBL-positive *E. coli* followed by group D (16.67%; 6/36), B1 (8.33%; 3/36), and B2 (5.56%; 2/36) as depicted in Table 4.

**Table 4:** **Characteristics of ESBL-producing ***E. coli*** strains (***n*** = 36) isolated from mastitic cows**.

***E. coli* isolates**	**Place of isolation**	**Phlyo-groups**	**β-lactamase genes**	ISCR1^*^	**IS*CR1* association with *bla* genes**	**Integron class 1**	*IntI1*-VR^**^ amplicons (bp)	**Gene Cassettes 5′CS -3′CS**	**GenBank accession numbers**	R/I^*^ phenotypes to other non β-lactam antibiotics
I-3	Inner Mongolia	D	TEM-1+SHV-1	+	+	+	2200	*dfrA1-aacA4*	KY114582	Cip; C; NAL; SXT; TE
I-4	Inner Mongolia	A	CTX-M-15	+	+	+	1700	*dfrA17-aadA5*	KY114583	Cip; C; G; NAL; SXT; TE
I-5	Inner Mongolia	D	CTX-M-15+TEM-1+SHV-1	−	−	+	2200	*dfrA1- aacA4*	KY114584	Cip; G; NAL; SXT; TE
I-6	Inner Mongolia	A	TEM-1	+	+	+	1700	*dfrA17-aadA5*	KY114585	Cip; C; G; NAL; SXT; TE
I-12	Inner Mongolia	B1	CTX-M-15+TEM-1	+	+	−	−	−	−	Cip; G; NAL; SXT; TE
I-14	Inner Mongolia	A	CTX-M-15+ SHV-1	+	+	+	1700	*dfrA17-aadA5*	KY114586	Cip; C; G; NAL; SXT; TE
I-17	Inner Mongolia	A	CTX-M-15	−	−	+	1700	*dfrA17-aadA5*	KY114587	Cip; G; NAL; SXT; TE
I-18	Inner Mongolia	A	CTX-M-15	+	+	−	−	−	−	Cip; G; NAL; SXT; TE
I-22	Inner Mongolia	D	CTX-M-15+SHV-1	+	+	+	−	−	−	Cip; G; NAL; SXT; TE
I-25	Inner Mongolia	A	CTX-M-15+TEM-1+SHV-1	−	−	+	1700	*dfrA17-aadA5*	KY114588	Cip; G; NAL; SXT; TE
I_1_-1	Inner Mongolia	A	CTX-M-14	+	+	+	1800	*dfrA17-aada4*	KY114589	−
I_1_-2	Inner Mongolia	A	TEM-1	+	+	+	−	−	−	G; TE
I_1_-3	Inner Mongolia	A	TEM-1	+	−	−	−	−	−	−
I_1_-4	Inner Mongolia	A	CTX-M-15	−	−	+	1700	*dfrA17-aadA5*	KY114590	−
I_1_-5	Inner Mongolia	A	CTX-M-15	_	_	_	_	_	_	NAL
I_1_-7	Inner Mongolia	B1	CTX-M-55+TEM-1	_	_	+	1700	*dfrA17-aadA5*	KY114591	Cip; C; G; NAL; SXT; TE
I_1_-8	Inner Mongolia	A	CTX-M-14	−	−	+	1800	*dfra17-aada4*	KY114592	C; G; NAL; SXT; TE
I_1_-11	Inner Mongolia	B1	TEM-1	−	−	+	1700	*dfrA17-aadA5*	KY114593	C; G; NAL; SXT; TE
I_2_-1	Inner Mongolia	A	CTX-M-15+TEM-1	−	−	+	1700	*dfrA17-aadA5*	KY114594	Cip; C; G; NAL; SXT; TE
I_2_-2	Inner Mongolia	A	TEM-1	−	−	+	1700	*dfrA17-aadA5*	KY114595	C; G; NAL; SXT; TE
I_2_-3	Inner Mongolia	A	CTX-M-14+TEM-1	−	−	+	1800	*dfra17-aada4*	KY114596	C; G; NAL; SXT; TE
I_3_-1	Inner Mongolia	B2	CTX-M-15	+	+	+	1700	*dfrA17-aadA5*	KY114597	C; G; NAL; SXT; TE
I_3_-2	Inner Mongolia	B2	CTX-M-15	+	+	+	1700	*dfrA17-aadA5*	KY114598	C; G; NAL; SXT; TE
G-1	Guangdong	A	CTX-M-15	+	+	+	1700	*dfrA17-aadA5*	KY114599	Cip; C; NAL; SXT; TE
G-2	Guangdong	A	CTX-M-15+TEM-1	+	+	+	1700	*dfrA17- aadA5*	KY114600	C; NAL; SXT; TE
J-4	Jiangsu	D	CTX-M-15+TEM-1	+	+	+	−	−	−	C; G; NAL; SXT; TE
J-5	Jiangsu	D	CTX-M-15+TEM-1	+	+	+	−	−	−	C; G; NAL; SXT; TE
Hb_2_-4	Hebei	D	TEM-1	−	−	+	2000	*dfrA17-aadA5*	KY114601	C; G; NAL; SXT; TE
Hb_3_-1	Hebei	A	CTX-M-15	+	+	+	1000	*aadA1*	KY114602	Cip; C; G; NAL; SXT; TE
L-1	Liaoning	A	CTX-M-15+TEM-1	+	+	+	1700	*dfrA17-aadA5*	KY114603	C; G; NAL; SXT; TE
N-1	Ningxia	A	CTX-M-3+TEM-1	−	−	+	1300	*aadA5*	KY114604	TE
H-5	Heilojinag	A	SHV-12	+	+	−	−	−	−	−
Hn-6	Henan	A	CTX-M-15	−	−	−	−	−	−	C; TE
Hn_1_-2	Henan	A	CTX-M-1	+	+	+	1700	*dfrA17-aadA5*	KY114605	G; NAL
Hn_1_-6	Henan	A	CTX-M-15+TEM-1	+	−	+	1700	*dfrA17-aadA5*	KY114606	G; NAL; SXT; TE
Hn_1_-7	Henan	A	CTX-M-15+TEM-1	+	+	+	1700	*dfrA17-aadA5*	KY114607	G; NAL; SXT; TE

R/I, Resistance/Intermediary; aacA4, aminoglycoside 6′-N-acetyltransferase; aadA, aminoglycoside adenyltransferase; dfrA1, dihydrofolate reductase type A; dfrA17, dihydrofolate reductase DHFRXVII; Cip, ciprofloxacin; C, chloramphenicol; G, gentamicin; NAL, nalidixic acid; SXT, trimethoprim/sulphamethoxazole; TE, tetracycline.

* ISCR1: Insertion sequence common region 1.

** IntI1-VR-: class 1 integrons variable regions, approximate size of base pairs deduced from running the amplicons on 1% agarose gel and sequencing the amplicon.

### Antibiotic susceptibility profiles

All 36 ESBLs-producing *E. coli* isolates were found to be multiple-drug resistant (MDR). However, different isolates exhibited slight variation in their antibiotic susceptibility profiles against the 16 tested antibiotics (Table 3). The majority of the isolates were resistant to first (cephalexin, 100%), second (cefaclor, 94.4%), third (cefotaxime and ceftazidime, 100% and 66.67%, respectively), and fourth (cefepime, 58.33%) generation cephalosporins. However, a high rate of susceptibility was observed toward cefamycin (cefoxatin, 83.34%) and carbapenem (meropenem, 100%), but susceptibility to monobactams (aztreonam, 13.89%) was low. The isolates were also resistant to other β-lactam and non-β-lactam antibiotics including ampicillin (88.89%), amoxicillin/clavulanic acid (75.00%), chloramphenicol (52.78%), ciprofloxacin (44.44%), gentamicin (72.22%), nalidixic acid (80.56%), tetracycline (83.33%) and trimethoprim/sulphamethoxazole (75%).

### Detection of integrons, gene cassettes and I*SCR1*

Thirty (83.33%) of the ESBL-producing *E. coli* carried clinical class 1 integrons but class 2 and class 3 integrons were not detected in any of the isolates. Among the *intI1*+ESBL-producing *E. coli*, 24 (80.00%) isolates tested positive for the presence of variable regions, while six of the isolates could not be amplified (Table 4). Furthermore, these 24 isolates were also positive for *qacE*Δ*1/sul1* indicating a complete clinical class 1 integron. Integrons lacking 3′CS were not PCR amplified for *qacE*Δ*1/sul1* (results not shown).

**Figure 3:**
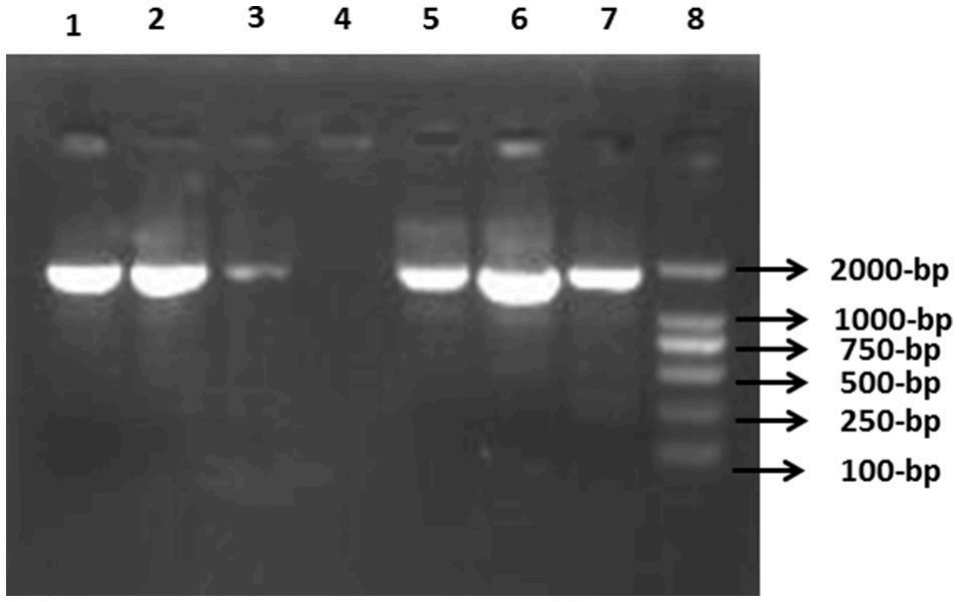
**Detection of class 1 integrons variable regions in ESBL-producing ***E. coli*****. PCR product was separated on 1% agarose gel. Lane 1, I-3 (*intI1*+) isolate; Lane 2, I_1_-8strain; Lane 3, G-2 isolate; Lane 4, H-5 (*intI1*-ve) isolate; Lane 5, I_2_-3 *E. coli*; Lane 6, Hn_1_-2 strain; Lane 7, positive control strain; Lane 8, 2K molecular marker (Transgen, Beijing, China).

**Figure 4:**
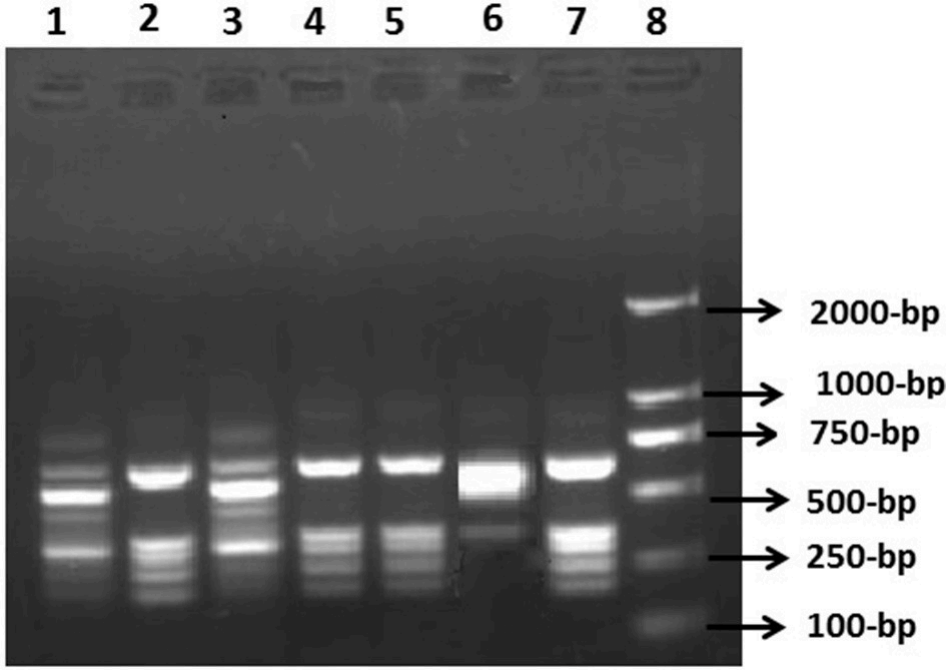
**Restriction fragment length polymorphism (RFLP) analysis of ***intI1*** variable region amplicons using ***Hinf I*** enzyme**. RFLP product was analyzed on 1.5% agarose gel. Lane 1, I-3 isolate; Lane 2, I-25 *E. coli*; Lane 3, Hn_1_-2 isolate; Lane 4, I_2_-3 isolate; Lane 5, G-2 strain; Lane 6, Hb_3_-1isolate; Lane 7, Hn_1_-7 *E. coli* strain; Lane 8, 2K molecular marker.

The PCR-amplicon sizes of the inserted gene cassettes ranged between ~1.0 and ~2.2 kb with the most predominant being ~1.7 kb amplicons (Figure 3). Most of the PCR amplicons of the variable regions of gene cassette arrays were a single band. However, two of the isolates produced a double band (of ~1.7 and ~ 0.2 kb). Subsequent sequence analysis of the gel extracted amplicons indicated that the smaller band was nonspecific amplification. Different band profiles of PCR-RFLP products indicated five distinct genotypic configurations (Figure 4). The most predominant PCR-RFLP genotype produced a profile of ~0.6, ~0.4, ~0.45, ~0.2, and ~0.22 kb restriction fragments consistent with digestion of 1.7 kb PCR amplicon of the variable regions. Amplicons sequence analysis of the variable regions revealed five gene cassettes carrying single or two genes in tandem. The predominant combination was *dfrA17-aadA5* in tandem that conferred resistance to aminoglycosides and trimethoprim. Interestingly, all the CTX-15-positive isolates, except two, carried *dfrA17-aadA5* genes in combination. This was consistent with the antibiotic susceptibility profile of these isolates reflecting resistance to the relevant drugs (Table 4). Surprisingly, no ESBL genes were found encoded in the variable region of the gene cassette array of these isolates. Therefore, IS*CR1* elements were investigated by targeted-PCR. The PCR amplicons of IS*CR1* elements were sequenced and confirmed by homology. Results indicated that IS*CR1* was found in 22 (66.11%) ESBL positive isolates (Table 4). Moreover, IS*CR1* (Accession number KY095113) was found associated with *bla*_CTX-*M*_, *bla*_TEM_ and *bla*_SHV_ in 16, 3, and 4 isolates, respectively. Interestingly, all *bla*_CTX-*M*-15_ positive isolates, except one (Hn1-6), have been always found associated with IS*CR1* elements. However, *bla*_TEM_, when found alone or in combination with others, except *bla*_CTX-*M*-15,_ was mainly negative for IS*CR1* elements. The amplicon size resulting from PCR using primer combinations specific to IS*CR1* elements and ESBL genes revealed that the ESBL genes were oriented downstream of IS*CR1* elements. Altogether, these results indicated that IS*CR1* elements are associated with ESBL genes. The detailed characterizations of all ESBLs-producing *E. coli* are elaborated in Table 4.

## Discussion

In the past few years, ESBL-producing *E. coli* have been increasingly isolated from food-producing animals raising global concerns for veterinary and public health (Seiffert et al., 2013). The current study reports on the higher occurrence (23.53%) of ESBL-producing *E. coli* (*n* = 36) among 153 *E. coli* isolates from mastitic cows, as compared to previous reports from China (Yu et al., 2015) and other countries (Dahmen et al., 2013; Geser et al., 2015; Freitag et al., 2016). Interestingly, the majority of ESBL-producing *E. coli* (23 isolates) were recovered from herds in Inner Mongolia province possibly linked to dense farming (2.37 million dairy cows) with the largest dairy herds in this province (Dou, 2014). Dense farming is significantly correlated with the incidence of bovine mastitis (Ali et al., 2014), and mastitis is the main reason for frequent and prolonged use of antibiotics that exert selective pressure for emergence and dissemination of resistant isolates (Berge et al., 2005). Our results revealed that CTX-M, mainly the CTX-M-15, was the most prevalent genotype, followed by TEM and SHV. These findings agree with other contemporary studies from China and around the world that also reported CTX-M as the dominant ESBL genotype (Locatelli et al., 2009; Dahmen et al., 2013; Geser et al., 2015; Kar et al., 2015). This goes along with the recent detection of CTX-M-15 producing *E. coli* from cattle and other food-animals in east Asia (Ohnishi et al., 2013; Yu et al., 2015), India (Upadhyay et al., 2015), the United Kingdom (Timofte et al., 2014), Germany (Freitag et al., 2016) and Tanzania (Seni et al., 2016). A national resistance surveillance study in China reported that the prevalence of ESBL-producing *E. coli* in humans has persisted above 50% since 2000 (Xiao et al., 2011), and recently Liu et al. reported even higher prevalence (68.2%) of ESBLs in clinical *E. coli* isolates, mainly the *bla*_CTX-*M*-15_ (Liu et al., 2015). Similarly, in animals the prevalence of ESBL-producing *E. coli* in China has considerably increased in recent years with CTX-M being the major prevailing gene encoding for ESBLs (Rao et al., 2014). It is known that, generally, ESBL genes are located on plasmids that could spread easily among commensal and pathogenic bacteria in the herd and the environment. Due to limited resources, in the present study, we could not investigate the prevalence of ESBL genes in other bacteria, in other healthy cows, or in cows with subclinical mastitis which is usually 30–40% higher than clinical mastitis (Halasa et al., 2007). We presume that the actual prevalence of ESBL-producers, particularly *E. coli*, may be much higher than the reported.

In ESBL-positive *E. coli*, phylogenetic group A represented the most prevalent group followed by virulent extra-intestinal group D; however, this was in contrast to our previous study (Liu et al., 2014), which reported that the pathogenic *E. coli* associated with mastitis mainly belonged to phylo-group B1 (58.6%) rather than group A (35.7%). Nevertheless, similar phylogenetic distributions were observed in some ESBL-producing *E. coli* isolated from animals (Abraham et al., 2014; Xu et al., 2015). Our results indicated that all the ESBL-producing *E. coli* isolates were MDR. The majority of these isolates (54–100%) were found resistant to cephalosporins. In addition, low susceptibility was also observed against the common β-lactam and non-β-lactam antibiotics such as ampicillin, aminoglycosides, tetracycline and fluoroquinolones. Recently, many studies have reported MDR ESBL-producing *E. coli* isolated from cattle (Timofte et al., 2014), poultry (Kar et al., 2015), pigs (Xu et al., 2015), and humans (Gu et al., 2008). Fluoroquinolones, following ciprofloxacin, are the second important antimicrobial drug in veterinary and human medicine (Coque et al., 2008). Quinolone resistance is traditionally caused by chromosomal mutations in gyrase or topoisomerase encoding genes or efflux pump expression regulating genes (Hopkins et al., 2005); nonetheless, plasmid mediated quinolone resistance is also increasingly reported in ESBL-producing *E. coli* (Xu et al., 2015).

Integrons play an important role in the emergence of MDR bacteria and in the dissemination of resistance genes. Published reports on the characterization of integrons in ESBLs-positive *E. coli* from dairy cows are scarce, but previous studies have been conducted in other food-animals, humans and the environment (Gu et al., 2008; Chen et al., 2010; Xu et al., 2015). In accordance to these studies, clinical class 1 integrons were found in the majority of ESBL-positive *E. coli* (83.33%). The gene cassette arrays of the class 1 integron variable regions contained five different gene combinations that likely impart additional resistance features to our isolates (see Table 3). Six of the *intI1* positive amplicons were failed to generate gene cassettes which may be related to the absence of 3′CS in these integrons (Lu et al., 2010). The *dfrA17-aadA5* was the predominant gene array that corroborates with the previous studies in China (Gu et al., 2008; Xu et al., 2015). Strikingly, we determined that majority of *bla*_CTX-*M*_ genes were associated with IS*CR1* elements, but no ESBL genes were found in the class 1 integron cassettes. It agrees with other published reports that also did not detect ESBL genes in the cassettes (Kiiru et al., 2013; Kar et al., 2015; Xu et al., 2015). Notably, our findings of the most pre-dominant CTX-M type (*bla*_CTXM-15)_ and its association with the IS*CR1* elements rather than gene cassette arrays indicated that they are more likely mobilized by IS*CR1* elements. Conversely, *bla*_TEM,_ when found alone or not associated with *bla*_CTXM-15_, was not often found linked to IS*CR1*, and therefore, was comparatively less prevalent. It has been proposed that antibiotic resistance gene elements are added to the 3′-CS of class 1 integrons by co-mobilization with the nearby IS*CR1* from the neighbor integron, implying rolling circle transposition and homologous recombination mechanisms, thus facilitating the formation of complex class 1 integrons (Toleman et al., 2006). Taken together, the current high occurrence of multi-resistant ESBL-producing *E. coli* carrying clinical class 1 integrons and its association with IS*CR1* is worrisome. This may suggest these bacteria are armouring against the antibiotics by devising various tools to render antibiotics useless. Fear exists that this co-existence of ESBL genes along with class 1 integrons as gene cassettes and I*SCR1* mobile elements would more robustly disseminate resistance elements within bacterial populations. This calls for an efficient control policy with restriction on the consumption of extended spectrum cephalosporins for long term use.

## Conclusions

Here, we report on the high occurrence of ESBL-producing *E. coli* from bovine mastitis. Genotypic characterization indicated a dominance of *bla*_CTX-*M*-15_ genes harboring clinical class 1 integrons associated with IS*CR1* elements, indicative rapid and wider dissemination potential and posing threats to veterinary and public health. To the best of our knowledge, this is the first comprehensive study to report on the alarming high prevalence of *bla*_CTX-*M*-15_ and class 1 integron resistance conferring elements in ESBL-producing *E. coli* from mastitic cows in China.

## Author contributions

BH, TA, and SR, conceived and designed the experiment. TA, MS, and SZ, performed the research. JG, GL, and LZ, contributed in reagents/materials/analysis. BH, TA, and SR, wrote the manuscript.

## Funding

This research was supported by the Chinese Twelfth “Five-year” National Science and Technology Support Project (No. 2012BAD12B03), Ministry of Education in China major project (No. 313054), Specialized Research Fund for the Doctoral Program of Higher Education (SRFDP) State Education Ministry (No. 20120008110042), and the National Natural Science Foundation of China (No. 3151101034) and (NO. 31572587).

### Conflict of interest statement

The authors declare that the research was conducted in the absence of any commercial or financial relationships that could be construed as a potential conflict of interest.
